# Twelve tips to combat ill-being during the COVID-19 pandemic: A guide for health professionals & educators

**DOI:** 10.15694/mep.2020.000070.1

**Published:** 2020-04-17

**Authors:** Adam Neufeld, Greg Malin

**Affiliations:** 1 University of Saskatchewan College of Medicine

**Keywords:** pandemic, COVID-19, ill-being, motivation, basic psychological needs

## Abstract

This article was migrated. The article was marked as recommended.

**Background:** Self-determination theory (SDT) represents an organismic theory of motivation and well-being, viewing people as naturally evolving creatures with innate needs for growth, mastery, and connection. According to SDT, for these tendencies to function optimally and for people to flourish, they require support of three basic psychological needs-autonomy, competence, and relatedness. During a pandemic such as the coronavirus disease 2019 (COVID-19), which can provoke isolation, fear, and feelings of helplessness, it is more important than ever to prioritize and support each other’s basic psychological needs.

**Aim:** The concept of basic psychological need satisfaction is relevant in the health professions, but during a crisis, it is easy for these needs to get overlooked or thrown aside. Through this article, we aim to make this concept more understandable and applicable by those in the health and education professions, including students.

**Methods:** SDT literature was foundational to creating these practical guidelines.

**Results:** The authors present 12 SDT-derived tips for practitioners, educators, administrators, and learners, on ways to engage in need-supportive behaviour and promote well-being during the COVID-19 pandemic.

**Conclusion:** These tips demonstrate that going back to the basics in times of emergency and stress can help optimize outcomes while fostering connection, ability, and purpose. They can be learned through practice and applied to anything, from emails and social media, to teaching, to patient care.

## Introduction

As the novel coronavirus disease 2019 (COVID-19) continues to spread, directly affecting everyone’s lives and testing the resilience of our health care systems, it is more important than ever to prioritize our health and well-being ( Lau *et al.*, 2008). Part of this means supporting our leaders in the health professions (practitioners, teachers, administrators, and students), who despite lacking the workforce to meet demands and being at risk for burnout, even before COVID-19, continue to sacrifice for the good of others ( Orsini, Binnie and Wilson, 2016a; Unadkat and Farquhar, 2020). As we prepare for what lies ahead, it is important to stick to the basics when supporting each other, both for maintaining our own and others’ well-being. This article uses self-determination theory (SDT) as a framework to provide guidance on this.

Backed by 30 years of research, SDT tells us that all human-beings require fulfilment of three basic psychological needs for optimal health, resilience, and well-being-autonomy, competence, and relatedness ( Ryan and Deci, 2017). Autonomy is our need for agency and to feel in control of our lives (as opposed to feeling controlled or helpless). Relatedness is our need to feel connected and that we matter to those around us, which is deeply satisfied when we feel respected and valued. Competence is our need to feel effective in what we do, which is fuelled by overcoming challenges. It helps buffer fatigue and burnout by revitalizing us and enabling us to tackle new challenges. Research shows that supporting these basic psychological needs is associated with a range of beneficial outcomes, including deeper commitment, greater mental toughness, increased productivity, and better overall coping and well-being ( Vansteenkiste and Ryan, 2013; Mahoney *et al.*, 2014; Ryan and Deci, 2017; Orsini, Binnie and Wilson, 2016a).

In his article on caring for caregivers during COVID-19 ( Rigby, 2020), SDT expert Scott Rigby describes how these essential human needs can often be mistaken for luxury ingredients (for when times are good), when in fact they are essential to heed in a time of crisis. In light of COVID-19, other articles have emerged which echo this sentiment-that SDT’s need satisfaction model can be used as a framework to help support peoples’ well-being during the pandemic, including doctors ( Teoh and Kinman, 2020; Parrish, 2020). As we come to grips with COVID-19 and the disruption it brings to our lives, let us remember and foster the basic nutriments we all need in common-autonomy, competence, and relatedness. The following 12 tips are outlined in relation to these basic needs (though many overlap) and how to support them during COVID-19. They are meant to provide a blueprint for ways that health professionals and educators can support each other and boost morale, performance, and well-being.

### Autonomy

#### Tip 1-Provide rational ‘why’ guidance

A pandemic like COVID-19 represents a chaotic time and demands systematic and timely adjustment, often requiring leaders to adopt a “command and control” style of leadership ( Snowden, 2010). This can be critical for disseminating timely information, and for creating the necessary sense of urgency people need to buy in. That said, using controlling (“these are the rules”) directives, without an adequate explanation, tends to undermine autonomy and lead to defiance. Without taking the time to provide rationale for decisions (i.e. when sending email updates, implementing policy changes, making requests of people, etc.), which is critical to supporting autonomy ( Deci and Ryan, 2008; Orsini, Binnie and Wilson, 2016b), people will simply not internalize the message. Whether with patients, employees, or students, doing so shows them they are worthy of it being explained to (which helps them feel like stakeholders) and lays the foundation for better engagement and commitment.

#### Tip 2-Take (and encourage) accountability

Another important consideration is that the vast majority of health professionals are trained to work in relatively stable environments (i.e. with predictable variables), so during a time of crisis, everyone is working from a place of relative inexperience. Everyone is feeling uncertain and learning how to cope, so whether you are a leader or staff or student, it is important to consider and acknowledge others’ perspectives in this time. This may be especially relevant to remote workers. For example, while institutions scramble to get the technology right, a common and unfortunate consequence can be to lose sight of virtual leadership and team dynamics. This can inadvertently lead managers to micromanage, time monitor, and use other controlling tactics to press for accountability-all which are counterproductive and undermine remote workers’ autonomous motivation ( Rajkumar *et al.,* 2016). It is therefore critical to trust in remote workers and their ability to self-direct, during these times. While this invariably requires more flexibility and open communication, empowering people and supporting their autonomy will better facilitate engagement and productivity, than imposing strict external monitoring ( Williams and Deci, 1998).

#### Tip 3-Re-invent ways to be autonomy-supportive

Working remotely during a pandemic can be exceptionally challenging. For example, telephone and computer interfaced communication can lead to poorer public and patient engagement; however, it can also bolster it, depending on what is emphasized ( O’Connor *et al.*, 2016). This is where fostering autonomy-support can pay dividends (i.e. presenting information factually and non-judgementally, actively engaging others while taking their perspectives and needs into account, and providing opportunities for choice) ( Williams and Deci, 2001; Williams *et al.*, 1999). While achieving this can be a little challenging even in the classroom, office, or clinic setting, with some strategic effort, supporting peoples’ autonomy can unlock more meaningful and productive exchanges. One way to do this for educators and administrators is to utilize various online platforms that enable group chat and video conferencing, since these promote participant discussion and offer choices for engagement. Similarly, as COVID-19 provokes policy changes in health care provision, health providers can successfully adapt their practices by using telemedicine or video conferencing ( Gibson *et al.*, 2011; Zhou *et al.*, 2020) and enrich the quality and outcomes of those interactions by focusing on providing autonomy-supportive patient care ( Rubeis, Schochow and Steger, 2018; Entwistle *et al.*, 2010).

#### Tip 4-Communicate mindfully

During a pandemic (e.g. COVID-19), when there are literally hour-by-hour updates and new public health policies being proposed and implemented, it can be challenging for leadership to convey actionable information without being harsh or controlling ( Gerwin, 2012). A key to achieving this is to try to remain transparent, forthcoming, and consistent (i.e. without overpromising anything, glossing over tough news, or changing positions without justification). To that end, using autonomy-supportive language (e.g. “can,” “may,” and “could,”) rather than controlling language (e.g. “must,” “need,” and “should”) can go a long way, towards optimizing the delivery and how well the message is received ( Kusurkar, Croiset and Ten Cate, 2011). Clearly, using procedural language may at times be necessary for public safety, but avoiding it where possible will improve the likelihood that people will accept direction. This is because autonomy-supportive language improves peoples’ ability to process what is happening, which tends to facilitate their ability to cope with stress ( Weinstein and Ryan, 2011). In turn, it tends to translate into them being more empathic toward others ( Williams and Deci, 1996).

### Competence

#### Tip 5-Reconnect with the purpose of supporting others

Although health professionals and educators may be well-positioned to help others and find purpose in their lives right now, during this time of stress and long work hours, it is good to reconnect with that purpose, and to not lose sight of it. Consider medical students, who are currently unable to engage in clinical work and who have to find that purpose through other means (e.g. volunteering). For inspiring examples of ways that medical students are responding to the COVID-19 pandemic and trying to support others, see the Canadian Medical Association’s website ( www.covidkindness.ca). For anyone whose volition and energy may be lacking, it may be helpful to get involved in side-projects, or to schedule additional telephone calls, e-meetings, or group chats (e.g. with team members, organization leaders, or with friends & family). Leaders would do well to try and support this in their faculty, staff, and learners.

#### Tip 6-Find structure in the uncertain

Let’s face it-during a pandemic, all of our routines are thrown off and the world can seem a pretty uncertain place. This can stunt people’s sense of motivation and halt momentum in its tracks. Suddenly, meeting deadlines, studying for exams, and even basic chores like cooking, cleaning, and personal hygiene can become difficult. While staying optimistic and keeping the big picture in mind is beneficial, so is breaking up your day into chunks and focusing on small goals, while taking appropriate breaks. Creating things to check off (e.g. cooking, cleaning, exercising, studying) can be surprisingly satisfying and help restore our purpose and self-determination. This can also help us accept our reality and transform grief or inertia into action-e.g. I can wash my hands to stay safe. I can start with a modest meal plan or ask for help with groceries. I can follow my normal bedtime and wake up routine, and schedule in exercise. I can live in the basement to protect my family. I can develop protocols to protect our teams in the hospital and care for patients through telemedicine. Setting these kinds of goals can bolster our health and well-being and help us persist during times of hardship.

#### Tip 7-Educate others with empathy

As a leader in health and education, many will look to you for guidance during a pandemic, such as COVID-19. While educating others may fall outside your work duties, it can significantly benefit others and help mitigate public health risks-especially if you notice others are misunderstanding (or ignoring) important regulations, such as with social distancing. The challenge is how to respectfully address other people without policing or causing defensiveness. According to SDT, which emphasizes motivation for change emerging from within the person (versus by external coercion), autonomy-support and basic need satisfaction are key players in this process ( Vansteenkiste and Sheldon, 2006). Basically, people do not tend to internalize values prescribed by others they do not feel connected to, feel pressured by, or that are too underly or overly challenging ( Patrick and Williams, 2012; Miller and Rollnick, 2012). Hence, reaching out to and inspiring change within others may best be accomplished through first acknowledging their views and readiness for change, followed by provision of information and/or feedback to help them master the content. Practically, this might mean leading with questions (rather than directives), challenging ideas in constructive ways that promote self-discovery (instead of telling them the answers) and creating opportunities for choices. One example of this might be posting an educational message through social media and including a few user-friendly links or resources. This gets your point across (which can inform and challenge peoples’ beliefs and actions), promotes self-reflection (since it minimizes confrontation), and supports peoples’ confidentiality and choices (e.g. to ignore it, engage in public or private discussion, or share with others, etc.).

#### Tip 8-Engage in activities that excite joy and interest

As we isolate in our homes and look for ways to stay engaged, it can be easy to forget about doing things for fun. Spoiler alert: binge watching Netflix will only subdue restlessness for so long.. Though a pandemic is surely a call-to-action that, for many, necessitates going into work-mode, it can also serve as a time for reflection-about the things that really matter to us, what our priorities, interests, and commitments are, and what brings joy to our lives. Whether it is listening to or making music, starting a new family tradition, creating art, reading, or writing, or mastering a new hobby-whatever it is you enjoy doing simply out of interest and joy-that may be what helps sustain your motivation and wellness the most. And, if you happen to be in a position to help others engage in their own autonomous interests and activities-be it colleagues, patients, friends, or family-you will be playing an instrumental role in helping them form their own sense of mastery, growth, and vitality ( Mouratidis *et al.*, 2011; Nix *et al.*, 1999).

### Relatedness

#### Tip 9-Distance physically but not socially

To help “flatten the curve” of COVID-19, new government policies are regulating that everyone stay at home and practice “social distancing.” This is obviously key for mitigating health risks, and by many is considered a privilege. However, being isolated against one’s will can wreak serious havoc on peoples’ psychological well-being. For some, satisfying our needs for human connection may simply translate to using more video technology (e.g. Zoom, FaceTime, WhatsApp), in place of emails or texts, which can be less interactive and need-fulfilling. Video technology can also double to facilitate quality curriculum delivery for teachers ( Rice and Mckendree, 2013), more productive meetings and workdays for educators, administrators, and remote workers (e.g. through team “huddles”) ( Johnson, 2018), and potentially better patient-care for those isolated in hospital (e.g. through virtual patient-doctor interactions, or sharing video conference calls with loved ones) ( Wind *et al.*, 2020).

#### Tip 10-Take care of your own basic needs

For many, the workplace provides an environment ripe for need satisfaction-offering challenges, opportunities for achievement, and camaraderie. When that is lacking (i.e. due to social distancing and working from home), it may be helpful for individuals to consider their own basic needs for motivation and well-being, and if they are struggling, make adjustments to rectify their new situation. Relatedly, people in the health professions believe very strongly in the value of helping others (many working relentlessly on the frontlines), often at the cost of their own health and basic needs. What is important to remember is that healthcare workers are terrified and vulnerable too, making it indispensable for them to prioritize self-care, to stick together, and to support each other’s basic needs ( Teoh and Kinman, 2020; Orsini, Binnie and Wilson, 2016b). While it is normal that many are not feeling “okay,” at this time, and it may not feel quite right to ask, “how are you?” or “are you okay?”, the value of reminding ourselves and each other to engage in self-care cannot be overstated. While this can often be a conflict for health education workers, you cannot pour water into another’s glass if your own is empty.

#### Tip 11-Utilize social media with care

Self-isolation will inevitably ramp up social media use and online communication, and this can be helpful for staying connected and informed during times of disaster ( Keim and Noji, 2011). Indeed, social networks provide immediate access to news articles, live feeds and video content, commentaries, and forums-all of which can cater to our basic needs and help guide our day-to-day actions ( Zhu and Chen, 2015). However, taking in more information does not necessarily give us more control over the uncontrollable, and sometimes we can lose the trees for the forest. As is already being seen with COVID-19 ( Depoux *et al.*, 2020), social media can also propagate misinformation, spur destructive arguments, and exacerbate stress and anxiety ( Akram and Kumar, 2017; Brooks, 2015; Vannucci, Flannery and Ohannessian, 2017). That said, during this stressful time, consider the person behind the screen. Social media can bring us together and help us satisfy our needs for wellness, or it can segregate us and thwart our basic needs-we get to determine the outcome.

#### Tip 12-Listen to hear, not to problem-solve

Acknowledging others’ thoughts and feelings is critical in a time like COVID-19. At a time when tension and stress are heightened, sometimes opinions will vastly differ and it can lead to frustration and anger. As a manager, teacher, practitioner, or learner, it is important not to get defensive, to take a breath, and to do our best to listen-not for the sake of simply solving a problem, but to support each other. According to SDT, when people feel heard, they feel less controlled and more connected, and it puts them in a better position to be creative, persistent, and to problem-solve ( Ryan and Deci, 2017). This may be especially relevant in pressure-filled work environments (e.g. hospitals), where it is easy to become distracted and others’ basic psychological needs can get overshadowed. At this time, when everyone is grieving some kind of loss-a loved one, a job, a graduation, or just some normalcy-it is important to lean on each other and take a few minutes to just listen ( Stroebe *et al.*, 1996). Although physicians and health care leaders may be trained to compartmentalize their emotional responses, the moral injury associated with COVID-19 may take weeks, months, or even years to process and overcome, and they need the support too ( Maunder *et al.*, 2008).

## Conclusions

Basic psychological need satisfaction is especially relevant during a pandemic such as COVID-19, when well-being and motivation stand to take a hit. The 12 tips mentioned in this article speak to these psychological needs and ways to support them. While they are written for health professionals (practitioners, teachers, administrators, and students) and are intended to help especially during the current pandemic (COVID-19), these hints largely deal with self-regulation, leadership dynamics, and communication methods that undermine or promote peoples’ basic needs. Hence, they demonstrate that most anyone can engage in need-supportive behaviour and leadership, which can enhance our morale and well-being, performance, and ability to withstand hardship-through feelings of autonomy, competence, and relatedness.

These 12 tips can be applied in numerous types of scenarios, depending on the context (e.g. teaching students, managing a department and communicating with staff, working in clinics, in hospital, or from home). They may lend themselves to small or large group settings (e.g. online group-based learning), interactive meetings (e.g. telemedicine, or remote work meetings), and for individuals, in isolated settings.

## Take Home Messages

During a pandemic such as COVID-19, let us remember and foster the basic needs we all share in common for optimal functioning and well-being-autonomy, competence, and relatedness. These essential human psychological needs can often be overlooked or thrown aside during times of hardship, when they may be most useful and beneficial to pay attention to.

## Notes On Contributors

Adam Neufeld, MSc is a graduating medical student at the University of Saskatchewan and incoming Family Medicine resident at the University of Calgary. His areas of interest are in medical education and positive psychology. https://orcid.org/0000-0003-2848-8100

Greg Malin, MD, PhD is a member of faculty in the Department of Academic Family Medicine at the University of Saskatchewan. His research interests are in medical education, self-determination, and well-being. https://orcid.org/0000-0001-5650-4353

## Declarations

The author has declared that there are no conflicts of interest.

## Ethics Statement

Ethics approval was not required for this type of manuscript.

## External Funding

This article has not had any External Funding
