# Supplementary material for: Twelve tips to combat ill-being during the COVID-19 pandemic: A guide for health professionals & educators
Source: MedEdPublish (2016). 2020 Apr 17;9:70. [Version 1] doi: 10.15694/mep.2020.000070.1 (PMC10697553; doi:10.15694/mep.2020.000070.1)
Supplement: Peer review history

# Reviewer report: Reviewer response for version 1

**DOI:** 10.21956/mep.20040.r30685

**Published:** 2020-05-22

**Authors:** Yingzi Huang

**Affiliations:** 1 The First Affiliated Hospital, Sun Yat-sen University

This review has been migrated. The reviewer awarded 5 stars out of 5 I enjoyed reading this article and I wished I read it earlier when I was going through lasting difficult situation- Spring Festival til now in China. I especially like tip 5-reconnect with the purpose of supporting others as purpose is always less about what you want and more about what you give, which gives you something to live for and' why' that drives one forward when things are really bad.And perhaps only a purposeful meaningful life can possibly lead to transcendence of life and death despite painful memories. And Tip-12 would be extremely helpful as well-listen to hear, not to problem-solve. I would add that it's not just about listening to others but also about telling stories proactively to others, as one can reflect and reframe what happened through others' perspectives and positive feedback in difficult circumstances, where redemption,growth, love and hope are needed more than ever. Many thanks again for such an inspiring article.

Reviewer Expertise:

NA

No decision status is available

# Reviewer report: Reviewer response for version 1

**DOI:** 10.21956/mep.20040.r30678

**Published:** 2020-04-27

**Authors:** P Ravi Shankar

**Affiliations:** 1 American International Medical University

This review has been migrated. The reviewer awarded 4 stars out of 5 This is an interesting write up on twelve tips to combat ill-being during the ongoing pandemic. The pandemic has placed severe restrictions on a number of aspects of daily living and has shattered the daily routine of most individuals globally. The authors base their recommendations on the self-determination theory (SDT). Providing reasons (the why) for different measures may be important. The problem with COVID-19 is that most measures do not have clear-cut evidence in support of them but still the reasoning behind these measures and their expected outcomes can be mentioned. The data is still developing and is often contradictory. Trusting individuals to be accountable is a good suggestion. Most are dealing with substantial disruptions to their life and working from environments which may not be conductive which has to be considered. Tip 6 is interesting and breaking your day into chunks and doing each chunk properly can be a way forward. I personally still wake up most days with a positive sense of energy but by the middle of the day this can often wane. Helping people become more autonomous and self-motivated is important. I have often thought and many others have commented that ‘social distancing’ may not be an appropriate term. We want people to stay physically distant but socially connected. Finding joy in every day things may enable you to get through your day with optimism. Medical educators, administrators and students will find this article to be of interest. The principles may also be of interest to the general reader.

Reviewer Expertise:

NA

No decision status is available

# Reviewer report: Reviewer response for version 1

**DOI:** 10.21956/mep.20040.r30680

**Published:** 2020-04-19

**Authors:** David Taylor

**Affiliations:** 1 Gulf Medical University, Ajman, UAE

This review has been migrated. The reviewer awarded 5 stars out of 5 This is a really well-written paper, which takes as its framework a clearly delineated theoretical approach (Self Determination Theory). In my eyes, this is a coherent approach to developing and encouraging self-care which will (or should) outlast the current pandemic. I found this a stimulating and delightful read. Thank you!

Reviewer Expertise:

NA

No decision status is available

# Reviewer report: Reviewer response for version 1

**DOI:** 10.21956/mep.20040.r30681

**Published:** 2020-04-18

**Authors:** Sarmishtha Ghosh

**Affiliations:** 1 International Medical University

This review has been migrated. The reviewer awarded 4 stars out of 5 The 12 tips have come at a very appropriate time when all of us - students, teachers, clinicians across the globe are trying to adjust to this sudden change in working condition. Even though we were all mentally prepared that online teaching-learning will take over the scene, but the "pandemic" induced insecurity and expectations have created such a negative frame of well being. Not everyone is capable of combating stress and maintaining a balanced life. The authors have very nicely explained each item based on psychological principles which give a strong grounding of using the tips.This article can be used by anyone in the workforce. This article will help them in restructuring their life and get the best out of the current situation.

Reviewer Expertise:

NA

No decision status is available

# Reviewer report: Reviewer response for version 1

**DOI:** 10.21956/mep.20040.r30682

**Published:** 2020-04-18

**Authors:** Trevor Gibbs

**Affiliations:** 1 AMEE

This review has been migrated. The reviewer awarded 5 stars out of 5 During the time of Covid-19 and for probably a lot longer after it is eventually over, there will be many paper written on the subject. It is my hope that many of these papers look at the psychological aspects of the pandemic; patients, students, carers and faculty alike. I suppose that we all have our own ways of dealing with stress, some aspects work well, some less so. This paper outlines 12 tips in keeping as well as possible, I couch that in those terms because I cannot see how anyone might not feel stress during these times. This is a well written paper which outlines 12 approaches, written in a very simple but clear way. I like the way that the authors not only add the positivity in many of the tips, but how they describe some of the drawbacks from some of the tips.I feel that this paper is appropriate for all of us!

Reviewer Expertise:

NA

No decision status is available

# Reviewer report: Reviewer response for version 1

**DOI:** 10.21956/mep.20040.r30683

**Published:** 2020-04-17

**Authors:** Felix Silwimba

**Affiliations:** 1 University of Lusaka

This review has been migrated. The reviewer awarded 5 stars out of 5 educative, counselling and timely to health professionals

Reviewer Expertise:

NA

No decision status is available

# Reviewer report: Reviewer response for version 1

**DOI:** 10.21956/mep.20040.r30679

**Published:** 2020-04-17

**Authors:** Balakrishnan Nair

**Affiliations:** 1 Centre for Medical Professional Development and University of Newcastle

This review has been migrated. The reviewer awarded 5 stars out of 5 I found this timely review to be very useful for my clinical and educator roles. Every tip is put in context when based on psychology. This article allowed me to reflect on my actions during the past few weeks.

Reviewer Expertise:

NA

No decision status is available

# Reviewer report: Reviewer response for version 1

**DOI:** 10.21956/mep.20040.r30684

**Published:** 2020-04-17

**Authors:** Judy McKimm

**Affiliations:** 1 Swansea University

This review has been migrated. The reviewer awarded 5 stars out of 5 I really enjoyed this valuable 12 tips and found the way it was written and the framework very relevant and accessible. I’d recommend this to anyone working with learners, colleagues and living and relating to others (friends and family) - in other words, all of us as humans!One point, I really don’t like the term ‘ill-being’ and I think Changing the title to say promoting wellbeing would attract a lot of readers, especially right nowA big thanks to the authors for this, just off for my daily news fix!!

Reviewer Expertise:

NA

No decision status is available
